# Relationships among neurocognition, symptoms and functioning in patients with schizophrenia: a path-analytic approach for associations at baseline and following 24 weeks of antipsychotic drug therapy

**DOI:** 10.1186/1471-244X-9-44

**Published:** 2009-07-14

**Authors:** Ilya A Lipkovich, Walter Deberdt, John G Csernansky, Bernard Sabbe, Richard SE Keefe, Sara Kollack-Walker

**Affiliations:** 1 Lilly Research Laboratories, Eli Lilly and Company, Lilly Corporate Center, Indianapolis, USA; 2 Eli Lilly Benelux, Eli Lilly and Company, Brussels, Belgium; 3 Department of Psychiatry and Behavioral Sciences, Northwestern University Feinberg School of Medicine, Chicago, USA; 4 CAPRI (Collaborative Antwerp Psychiatric Research Institute), Department of Psychiatry, University of Antwerp, Antwerp B-2610, Antwerpen, Belgium; 5 Department of Psychiatry and Behavioral Sciences, Duke University Medical Center, Durham, USA; 6 Lilly Research Laboratories, Eli Lilly and Company, Lilly Corporate Center, Indianapolis, USA

## Abstract

**Background:**

Neurocognitive impairment and psychiatric symptoms have been associated with deficits in psychosocial and occupational functioning in patients with schizophrenia. This post-hoc analysis evaluates the relationships among cognition, psychopathology, and psychosocial functioning in patients with schizophrenia at baseline and following sustained treatment with antipsychotic drugs.

**Methods:**

Data were obtained from a clinical trial assessing the cognitive effects of selected antipsychotic drugs in patients with schizophrenia. Patients were randomly assigned to 24 weeks of treatment with olanzapine (n = 159), risperidone (n = 158), or haloperidol (n = 97). Psychosocial functioning was assessed with the Heinrichs-Carpenter Quality of Life Scale [QLS], cognition with a standard battery of neurocognitive tests; and psychiatric symptoms with the Positive and Negative Syndrome Scale [PANSS]. A path-analytic approach was used to evaluate the effects of changes in cognitive functioning on subdomains of quality of life, and to determine whether such effects were direct or mediated via changes in psychiatric symptoms.

**Results:**

At baseline, processing speed affected functioning mainly indirectly via negative symptoms. Positive symptoms also affected functioning at baseline although independent of cognition. At 24 weeks, changes in processing speed affected changes in functioning both directly and indirectly via PANSS negative subscale scores. Positive symptoms no longer contributed to the path-analytic models. Although a consistent relationship was observed between processing speed and the 3 functional domains, variation existed as to whether the paths were direct and/or indirect. Working memory and verbal memory did not significantly contribute to any of the path-analytic models studied.

**Conclusion:**

Processing speed demonstrated direct and indirect effects via negative symptoms on three domains of functioning as measured by the QLS at baseline and following 24 weeks of antipsychotic treatment.

## Background

Neurocognitive impairment has been found to be strongly correlated with deficits in psychosocial and occupational functioning in patients with schizophrenia [1,2]. These earlier reviews of the literature (including a meta-analysis) were focused on identifying specific neurocognitive deficits that restrict the functioning of schizophrenia patients, as opposed to the use of more global measures of neurocognitive functioning such as IQ. The results suggested associations between community functioning and verbal memory, card sorting/executive function, and verbal fluency, and associations between social problem-solving skills and vigilance and secondary verbal memory. Since these earlier reviews, a substantial body of research has confirmed and extended these observations.

Psychiatric symptoms have also been associated with functional impairment with early studies reporting the most consistent or strongest association for negative symptoms [3-5]. A substantial body of literature has since accumulated addressing, in part, the relative strength of associations of psychopathology and neurocognition with functional outcomes, with some studies reporting a greater effect of psychiatric symptoms on functioning [6,7], others concluding a greater role of neurocognition [1,8], and still others showing an important role of both [9-12]. The complexity among these findings, and in this area of research in general, likely reflects a number of moderating variables including patients' chronicity of illness, acute exacerbation versus more residual (stable) symptoms, overall symptom profile, study design (i.e., cross-sectional vs. longitudinal, inpatient/outpatient), and perhaps variability and redundancy across the plethora of psychometric tests (and component factor analyses) used to assess neurocognition, symptoms and functioning. Additional research is needed to understand how neurocognition and psychiatric symptoms can influence functional outcomes given the observed complexities and with the important goal of identifying those factors that can enhance patients' psychosocial and occupational functioning.

Several path-analytic studies have been conducted to evaluate the causal relationships among neurocognition, psychiatric symptoms, and functioning [8,13-15]. Velligan and colleagues reported a path model in which cognition predicted both concurrent symptomatology and activities of daily living while symptoms had little direct impact upon activities of daily living [8]. In patients switched from one antipsychotic to another (ziprasidone), Harvey presented a path analysis showing that improvement on the PANSS cognitive subscale directly affected changes on the PANSS anxiety-depression cluster and a "PANSS prosocial" subscale composed of items related to social engagement, while improvement on the PANSS anxiety-depression cluster had a direct effect on PANSS prosocial subscale [13]. Bowie and colleagues utilizing a composite score of neurocognition [14] reported that neuropsychological performance predicted functional capacity, which predicted three domains of real-world functioning (i.e., interpersonal skills, work skills, and community activities). In addition, depression predicted interpersonal and work skills, while negative symptoms affected interpersonal skills. Subsequently, Bowie and colleagues utilizing specific neuropsychological measures [15] reported that four cognitive factors (i.e., attention/working memory, processing speed, verbal memory and executive functioning) demonstrated both direct and indirect effects via functional competence and/or social competence on real world outcomes. Symptoms (positive, negative and depression) were directly related to outcomes, with fewer indirect relationships associated with the competence factors. Most of these studies suggest a role for both neurocognition and symptoms in mediating functional outcomes, although the models differ somewhat in the proposed relationship among the variables. Moreover, with the exception of the Bowie et al. article [15], most of these studies utilized composite measures to assess cognitive abilities and/or functioning.

Two recent studies assessed the magnitude of associations of psychiatric symptoms and neurocognition with the Heinrich's Quality of Life Scale (QLS) [11.12]. In the current study, we were interested in addressing potential causal relationships among specific neurocognitive domains – working memory, verbal memory, and processing speed, and discrete domains of functioning of the QLS – instrumental, intrapsychic, and interpersonal, at baseline and following 24 weeks of antipsychotic drug treatment in patients with schizophrenia. We used a path analytic approach to evaluate both the direct effects of neurocognitive domains on various aspects of patients' functioning, as well as the indirect effects of such domains, as mediated via changes in psychiatric symptoms. We were interested in evaluating these relationships prior to treatment and after treatment.

## Methods

### Subjects

Patients were enrolled in a 1-year, double-blind, randomized study of the neurocognitive efficacy of olanzapine [OLZ], risperidone [RIS], and haloperidol [HAL] in the treatment of schizophrenia [16]. Enrollment criteria included: patients 18 to 55 years of age who met the criteria for schizophrenia or schizoaffective disorder as defined by the *Diagnostic and Statistical Manual of Mental Disorders*, Fourth Edition [DSM-IV]. Patients (N = 414) were randomly assigned (1:1:1 ratio) to 52 weeks of treatment with OLZ (N = 159; 5 to 20 mg/day), RIS (N = 158; 2 to 10 mg/day), or HAL (N = 97; 2 to 19 mg/day). During the initial 8 weeks, a flexible dosing schedule was allowed; thereafter a fixed dose based on investigator's judgment was suggested. In this analysis, patients from all 3 treatment groups were included. As noted in the original report, the mean modal dose was 12.3 mg/day for olanzapine, 5.2 mg/day for risperidone, and 8.2 mg/day for haloperidol among patients who completed the study [16].

### Variables Assessed

Functioning was assessed with the Heinrichs-Carpenter Quality of Life Scale [QLS] [17]; we focused on 3 of the 4 subdomains of functioning. The first subdomain used was the Instrumental Role Functioning [QLS Instrumental], which measures the level of, and satisfaction with, occupational role functioning. We averaged the following 4 items: extent of occupational role functioning, level of accomplishment, degree of underemployment, and satisfaction with occupational role functioning. The second subdomain used was the Intrapsychic Foundation [QLS Intrapsychic], which measures variables related to sense of purpose, motivation, and empathy. We averaged the following 7 items: sense of purpose, degree of motivation, curiosity, anhedonia, time utilization, capacity for empathy, and capacity for engagement and interaction. The final subdomain used was the Interpersonal Relations [QLS Interpersonal], which measures the qualitative aspects of interpersonal relationships. We averaged the following 8 items: interpersonal relationship with household members, intimate relationships, active acquaintances, level of social activity, involved social network, social initiatives, and social withdrawal socio-sexual relations.

Psychiatric symptoms were assessed using the Positive and Negative Syndrome Scale [PANSS] [18]. This analysis focused on the PANSS overall score, and negative and positive subscale scores: PANSS overall = mean of 30 items [score of each item ranging from 1 (least severe) to 7 (most severe)], PANSS negative subscale [PANSS Neg] = mean of 7 items (#8, 9, 10, 11, 13, 21, 30), PANSS positive subscale [PANSS Pos] = mean of 8 items (#1, 3, 5, 6, 14, 15, 23, 26).

Patients were assessed with a comprehensive battery of neurocognitive tests [16]. We selected measures representing 3 major domains of cognition that we considered may be relevant as predictors of functional outcomes. As a sensitivity check, we considered incorporating a fourth "problem solving" or "executive" domain. In the presence of other cognitive variables, however, it did not add value in modeling changes in functional outcomes (via direct or indirect effects).

Cognitive variables were transformed into z-scores against healthy controls for 3 main areas of cognition: 1) working memory, as assessed by the Letter-Number Sequencing verbal subtest of the Wechsler Adult Intelligence Scale, Third Edition [WAIS-III]; 2) verbal memory, as assessed by the Rey Auditory-Verbal Learning Test with Crawford Alternative (10 minute); and 3) processing speed, computed as an average of the following 2 subscales: a) processing speed (digit-symbol coding), as assessed by the WAIS-R Digit-Symbol Coding performance subtest, and b) verbal fluency scale, constructed as an average of Category Instances and Controlled Oral Association Test. Although digit-symbol and verbal fluency are complex tasks that draw on a variety of cognitive processes, we followed the conceptualization of the MATRICS group, who placed verbal fluency and digit-symbol together in the same domain of "processing speed" based upon their review of several factor analyses available at the time [19]. Data from the Grooved Pegboard Tests were excluded from assessment of processing speed because of a large number of unusually high values.

### Statistical Methodology

Separate path-analytic models were evaluated for: 1) the 3 subdomains of functioning from the QLS – QLS Instrumental, QLS Intrapsychic, and QLS Interpersonal, and 2) relationships among pretreatment (baseline) measures and for changes in these measures to the 24-week endpoint. Therefore, a total of 6 models were constructed. Endpoint was defined as last observation prior to, or at, 24 weeks. We used a 24-week endpoint as significant changes were observed in both cognitive and functional measures at this time, and a substantial reduction in sample size had occurred by the next scheduled assessment (52 weeks).

**Figure 1:**
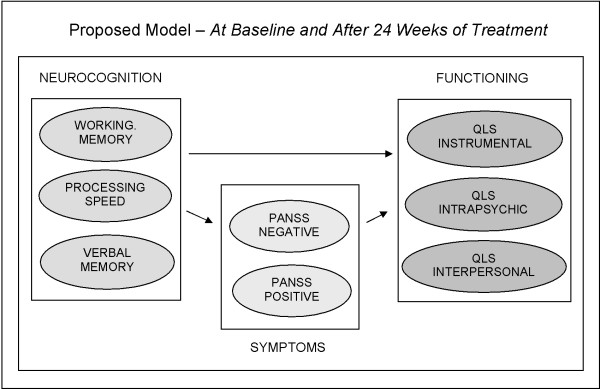
**In constructing path models, our fundamental assumption was that cognitive impairment precedes psychiatric symptoms and both precede functional impairment; therefore, cognitive status at baseline and changes in cognition may affect functioning either directly or indirectly via psychiatric symptoms**.

In modeling these relationships, our fundamental assumption was that cognitive impairment precedes psychiatric symptoms, and that both precede functional impairment as the illness of schizophrenia evolves in patients. Therefore, we hypothesized that cognitive status at baseline and subsequent changes in cognition could predict functioning either directly or indirectly via psychiatric symptoms (Figure 1), but not the other way around (e.g. changes in psychiatric symptoms affecting functioning via changes in cognition). Considering the various domains of psychopathology, we also hypothesized that negative symptoms would most strongly mediate the effects of cognition on functioning.

Each model incorporated the following: 1) 3 measures of cognition, including working memory, processing speed, and verbal memory as the independent variables; 2) PANSS Neg and PANSS Pos as intermediate outcomes; and 3) 1 of 3 functional domains, including QLS Instrumental, QLS Intrapsychic, and QLS Interpersonal as the dependent (outcome) measure.

For each outcome, we started with a model that was close to saturated and then excluded pathways that were not statistically significant and did not contribute to the overall model fit as measured by 3 criteria: 1) chi-squared statistics for the goodness of fit and associated p-value (significant p-value indicating poor fit); 2) Comparative Fit Index [CFI] (ranging from 0 to 1, higher values indicating better fit); and 3) Root Mean Square Error of Approximation [RMSEA]. An RMSEA of 0.05 or less indicates a good fit and an RMSEA of 0.10 or more indicates a poor fit.

Analyses were conducted using SAS^® ^Version 8 for PC. Path-analytic models were evaluated using SAS PROC CALIS.

## Results

### Patient Characteristics

The baseline values for all measures included in this analysis can be found in Table 1. The majority of patients were male with an average age of approximately 39 years of age. The average age of onset of the disease was approximately 23.1 years of age, with a mean number of 7 episodes. Averaged scores are provided for all of the scales assessed.

**Table 1:** Baseline characteristics.

***Demographics***	N = 395*
Gender (% females)	28.9%

Age, years; mean (SD)	39.1 (8.2)

Age at onset of disease, years; mean (SD)	23.1 (7.1)

***Severity of Psychopathology***	

Previous number of episodes; mean (SD)	6.65 (8.3)

PANSS overall score; mean (SD)	2.76 (0.46)

PANSS negative; mean (SD)	2.83 (0.81)

PANSS positive; mean (SD)	3.18 (0.66)

***Quality of Life Functional Domains***	

QLS Instrumental Role Functioning; mean (SD)	3.35 (0.90)

QLS Interpersonal Relations; mean (SD)	2.57 (1.16)

QLS Intrapsychic Foundation; mean (SD)	2.99 (1.13)

***Cognitive Subdomains (z scores against healthy controls)***	

Working Memory; mean (SD)	-1.01 (1.12)

Verbal Memory; mean (SD)	-1.41 (1.3)

Processing Speed; mean (SD)	-1.12 (0.80)

*Number based on patients with non-missing values for scales assessed.

### Changes in Cognitive Measures and Functioning from Baseline to Week 24

**Figure 2:**
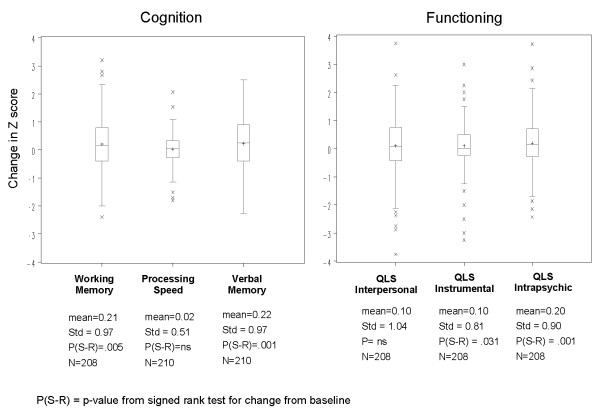
**Changes in cognitive measures and functioning from baseline to Week 24**.

Changes in cognitive measures and functioning from baseline are provided in Figure 2. For cognition, significant improvement from baseline was observed in working memory and verbal memory, although not in processing speed. For functioning, significant improvement from baseline was observed for the QLS Instrumental and QLS Intrapsychic subdomains, but not for the QLS Interpersonal subdomain.

### Path Analysis for Cognition, Symptoms, and Functioning at Baseline

**Figure 3:**
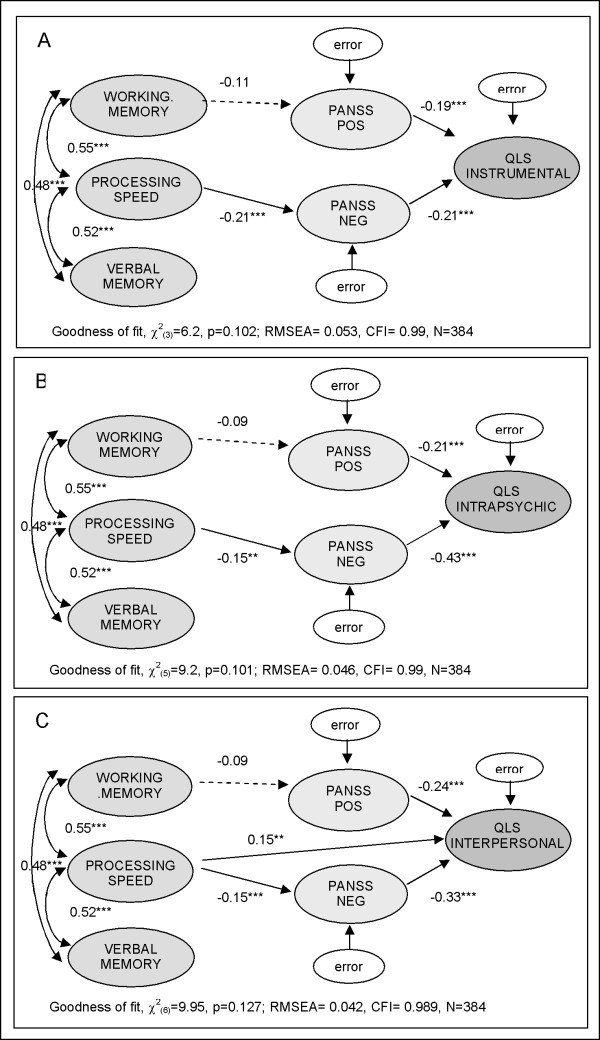
**Path diagram for relationships among cognition, symptoms, and occupational functioning at baseline for the 3 functional domains**: 1) QLS Instrumental, 2) QLS Intrapsychic, and 3) QLS Interpersonal. Values associated with directed pathways are standardized path coefficients; values over the double-arrowed arches are correlations; asterisks indicate statistical significance at *p < .05, **p < .01, ***p < .001. The pathways with z-scores less than 1.8 were not shown.

Pairwise correlations for measures at baseline are shown in Table 2. Inspection of these raw correlations suggests that selected cognitive measures and symptoms are highly correlated with certain domains of functioning. The direct and indirect relationships observed among cognition, symptoms and the three subdomains of functioning at baseline are illustrated in the path analytic diagrams presented in Figure 3.

**Table 2:** Pairwise Pearson correlations for measures at baseline (N = 395).

	QLS Intrapsychic	QLS Intpersonal	Processing Speed	Working Memory	Verbal Memory	Neg	Pos	PANSS Overall
QLS Instrumental	0.58***	0.47***	0.15**	0.13**	0.16**	-0.23***	-0.22***	-0.32***

QLS Intrapsychic		0.64***	0.21***	0.16**	0.15**	-0.48***	-0.26***	-0.47***

QLS Interpersonal			0.15**	0.02	0.02	-0.38***	-0.26***	-0.37***

Processing Speed				0.55***	0.53***	-0.16**	-0.03	-0.08

Working Memory					0.48***	-0.02	-0.09	-0.10

Verbal Memory						-0.07	-0.02	-0.11*

PANSS Neg							0.13*	0.61***

PANSS Pos								0.72***

*p < .05, **p < .01, ***p < .001

At baseline, processing speed affected functioning mainly indirectly via negative symptoms (PANSS Neg); patients who had faster processing speed had fewer negative symptoms and better overall functioning. Working memory and verbal memory did not significantly contribute to the path-analytic models at baseline. Positive symptoms (PANSS Pos) also contributed to the patient's functional status at baseline, independent of cognition.

### Path Analysis for Changes in Cognition, Symptoms, and Functioning at 24-Week Endpoint

**Figure 4:**
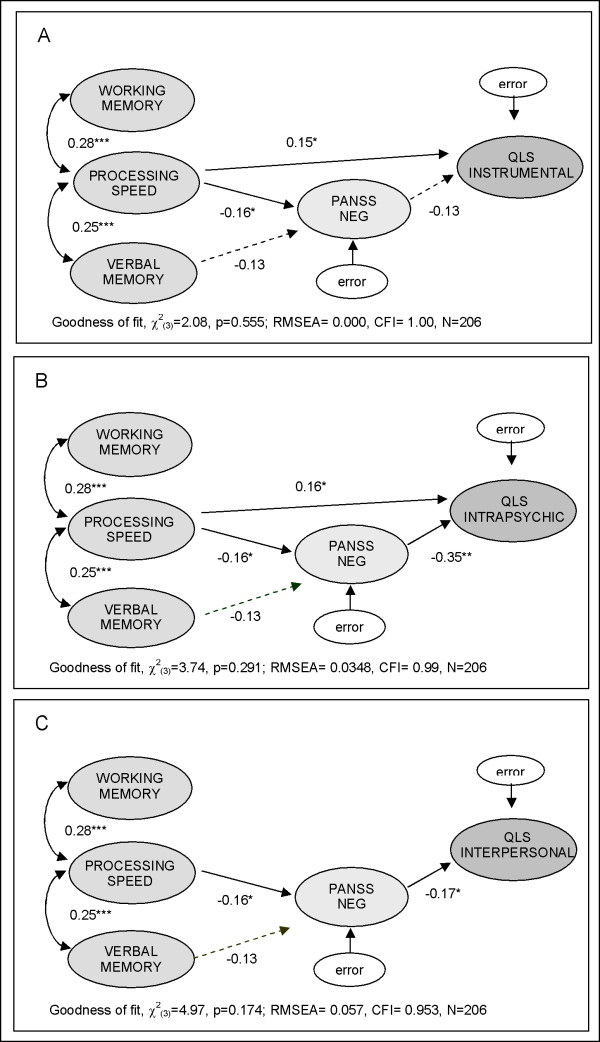
**Path diagram for relationships among changes in cognition, symptoms, and occupational functioning at 24 weeks for the 3 functional domains**: 1) QLS Instrumental, 2) QLS Intrapsychic, and 3) QLS Interpersonal. Values associated with directed pathways are standardized path coefficients; values over the double-arrowed arches are correlations; asterisks indicate statistical significance at *p < .05, **p < .01, ***p < .001. The pathways with z-scores less than 1.8 were not shown.

Pairwise correlations between changes in all measures at endpoint (Week 24) are shown in Table 3. Interestingly, these correlations were more moderate compared with those at baseline. Among cognitive measures, only changes in processing speed were significantly related to changes in functioning. Among symptoms, only changes in negative symptoms were significantly associated with changes in functioning. The path-analytic diagrams for the relationships among cognition, symptoms, and the 3 subdomains of functioning at 24 weeks are shown in Figure 4.

**Table 3:** Pairwise Pearson correlations between measures for changes to Week 24 (N = 208).

	QLS Intrapsychic	QLS Interpersonal	Processing Speed	Working Memory	Verbal Memory	Neg	Pos	PANSS Overall
QLS Instrumental	0.33***	0.23***	0.17*	0.06	0.03	-0.16*	-0.07	-0.14*

QLS Intrapsychic		0.46***	0.22**	0.11	0.06	-0.38***	-0.02	-0.17*

QLS Interpersonal			0.04	-0.11	-0.01	-0.17*	-0.09	-0.16*

Processing Speed				0.29***	0.25***	-0.17*	0.06	-0.05

Working Memory					0.08	0.04	-0.02	0.04

Verbal Memory						-0.15*	0.12	-0.05

PANSS Neg							0.12	0.60***

PANSS Pos								0.75***

*p < .05, **p < .01, ***p < .001

At 24 weeks, changes in processing speed affected changes in functioning, both directly and indirectly via changes in negative symptoms (PANSS Neg). Changes in working memory and verbal memory did not significantly contribute to the path-analytic models at 24 weeks. Changes in positive symptoms (PANSS Pos) were not significantly associated with changes in any of the 3 functional domains.

## Discussion

In this study, we evaluated the interaction among 3 neurocognitive domains (i.e., working memory, verbal memory, and processing speed) and 3 discrete domains of functioning (i.e., QLS Instrumental, QLS Intrapsychic, and QLS Interpersonal) at baseline and following 24 weeks of antipsychotic drug therapy in patients with schizophrenia. In our path-analytic models, we also incorporated positive and negative symptoms to assess whether cognitive variables may directly or indirectly affect functioning; in the latter case, via changes in symptoms. We found that only processing speed was significantly associated with functioning at both baseline and at 24 weeks. Processing speed affected functioning both directly and indirectly via negative symptoms, although the partitioning of the total effect into direct and indirect parts varied somewhat for each domain of functioning assessed, including QLS Instrumental (work), QLS Intrapsychic, and QLS Interpersonal (psychosocial) domains. In addition, we found that positive symptoms also affected functioning at baseline.

As reviewed by Bowie et al. [15], processing speed impairments may reflect a core cognitive deficit in schizophrenia. In a recent meta-analytic study, Dickinson and colleagues [20] reported that the digit-symbol coding task, a measure of processing speed, represented the greatest deficit among cognitive abilities in patients with schizophrenia. Processing speed was also identified as the most sensitive index in patients with schizophrenia for the WAIS-III technical manual [21]. Processing speed, as measured by the digit-symbol coding task, accounted for 65% of the variance in overall cognitive performance and was the best single predictor of total score in the Clinical Antipsychotic Trials of Intervention Effectiveness (CATIE) analyses [22]. Bowie and colleagues [15] found that the processing speed factor, which included the digit-symbol coding task, consistently predicted social competence and living skills and was the only factor to have a direct effect on all 3 real-world behaviors. It has been suggested that slowed information processing (including slowed encoding) can account for deficits in a range of higher level cognitive functions in schizophrenia including deficits in working memory, executive function and memory [23,24].

In the current study, we focused on the digit-symbol coding task and verbal fluency as two measures of processing speed. We were unable to utilize data from the Grooved Pegboard Tests because there were a large number of unusually high values. However, a recent review on psychomotor slowing in schizophrenia has suggested that psychomotor slowing may be distinguishable from a reduction in the speed of information processing (reviewed in [25]). While a number of neurocognitive processes that support motor control are likely involved in psychomotor functioning, in fact some tasks may be more sensitive to psychomotor speed while others are more sensitive to the speed of information processing. In this regard, the digit-symbol coding task would be more sensitive to the speed of information processing while the pegboard task would have been more sensitive to psychomotor speed.

Based on our proposed model, processing speed worked, in part, through negative symptoms to impact functioning. As proposed by Green and Nuechterlein [26], it is possible that the relationship between negative symptoms and functioning may reflect the shared variance between negative symptoms and neurocognition, or the stronger association between neurocognition and functional outcomes. There is some data to suggest that while cognitive deficits appear to develop at an earlier age than negative symptoms, some neurocognitive deficits may act as vulnerability factors for development of negative symptoms (reviewed in [26]). However, negative symptoms typically have shown relatively weak correlations to cognitive deficits [26], a finding that suggests that while there is some shared variance in predicting outcomes, negative symptoms and neurocognitive deficits are relatively distinct constructs. Thus, negative symptoms may also have a direct impact on a patient's overall level of functioning.

It has also been proposed that the causal pathways from negative symptoms to functional outcomes may be due to a common neurocognitive intersection [26]. *Does processing speed possibly serve as a neurocognitive intersection between negative symptoms and functioning? *Hughes and colleagues [27] reported that significant improvements in symptom ratings over time in patients with chronic schizophrenia did not predict improvements in any aspect of cognitive functioning measured, except motor speed. Rodriguez-Sanchez and colleagues [28] analyzed the relationship between different cognitive tasks and clinical symptoms in patients with first-episode schizophrenia and found that negative symptoms were significantly associated with performance on executive functions and motor coordination tasks, with a significant association of negative symptoms seen only for those executive functions requiring speeded performance. They concluded that the widely described relationship between negative symptoms and executive impairments in schizophrenia appears to be mediated by dysfunction in processing speed.

Some have argued that the Heinrichs-Carpenter QLS subtitled as "an instrument for rating the schizophrenia deficit syndrome" provides a clinical assessment of negative symptoms more than a subjective measure of a patient's quality of life [29]. Although not without controversy [30], we cannot exclude the possibility that the strong support for negative symptoms as a mediator between cognition and functioning may be somewhat unique to the use of the QLS.

In contrast to the Bowie et al. study [15], our path-analytic models did not suggest the presence of a significant relationship between working memory or verbal memory and functioning. However, previous work has incorporated additional constructs, such as social competence and functional competence, which may mediate the effect of other cognitive parameters, such as memory, on functioning [15,31].

The observation that positive symptoms affected functioning at baseline, but not after 24 weeks of treatment, was somewhat surprising. However, positive symptoms can improve quite rapidly with antipsychotic medication, and it is possible that the timing of improvement in positive symptoms may have occurred more quickly than improvement in functioning. Negative symptoms at baseline (and with change) appeared to play a more dominant role in functional outcomes.

Our inability to detect a stronger relationship between cognition and functioning may have been hindered by the lack of substantial improvement in both functioning and cognition observed during the 24-week time period that was analyzed. Long-term data on cognition and functioning are needed to obtain better estimation of potential direct and indirect effects of cognition on functioning of patients with schizophrenia.

A large number of dropouts (especially when caused by lack of symptom improvement) may have introduced bias in evaluating associations among changes in symptoms, cognition, and functioning at the 24-week endpoint. In the original report, 95 (59.7%) olanzapine-, 104 (65.8%) risperidone-, and 70 (72.2%) haloperidol-treated patients had discontinued for any reason; out of these, 20 (12.6%) olanzapine-, 18 (11.4%) risperidone-, and 16 (16.5%) haloperidol-treated patients had discontinued the study for lack of efficacy [16].

To evaluate whether dropouts could have biased the path analytic models estimated from observed changes at 24-week endpoint we performed a sensitivity analysis by imputing missing outcomes for subjects who discontinued prior to week 24 using a single imputation from a posterior predictive distribution of missing values given all the subjects' outcome data observed up to their dropout (with Bayesian regression for monotone missing data in SAS PROC MI). The results (not reported here) were qualitatively similar to those based on observed data at week 24.

While the relationship among variables could vary by treatment, analyzing the data separately by treatment groups would result in small subgroups with limited power to detect significant differences. In the original study [16], at the 52-week endpoint, neurocognition had significantly improved in each group, with no significant differences observed between groups. Although differences were observed on what specific domains improved in the three treatment arms, the lack of differences observed across the treatment groups overall and discontinuation of the haloperidol arm per protocol limit further analysis of this important issue in the current study.

Lastly, some studies have suggested that the effects of neurocognition are mediated primarily through social cognition and subsequently through social competence and social support [15,31]. We did not have data in which to assess the role of social cognition or other social variables in functioning.

## Conclusion

Processing speed demonstrated direct and indirect effects via negative symptoms on three domains of functioning as measured by the QLS at baseline and following 24 weeks of antipsychotic treatment. These results highlight the importance of improvement in negative symptoms and cognition (particularly processing speed) for improving functional outcomes of patients with schizophrenia.

## Competing interests

Drs. Lipkovich, Deberdt, and Kollack-Walker are employees of Eli Lilly and Company. In recent years, Dr. Sabbe received educational/research grants from AstraZeneca, Eli Lilly, Johnson & Johnson, Lundbeck and Sanofi-Synthélabo. He was a member of the scientific board of Bristol Myers Squibb, Eli Lilly, Glaxo Smith Kline, Johnson & Johnson, Novartis Pharma, Nycomed Belgium. Dr. Csernansky has provided consulting services to Eli Lilly, Sanofi-Aventis, and HoustonPharma. Dr. Keefe has received grant/research support from Astra-Zeneca and Eli Lilly, and NIMH. He has also served as a consultant and on advisory boards for various pharmaceutical companies as follows: Abbott Pharmaceuticals (advisory board), Acadia (consultant), BiolineRx (consultant), Bristol Myers Squibb (consultant), Cephalon (consultant), Cortex (consultant), Dainippon Sumitomo Pharma. (consultant), Eli Lilly (advisory board, consultant, speaker), Johnson & Johnson (consultant), Lundbeck (consultant), Memory Pharmaceuticals (advisory board, consultant), Merck (advisory board, consultant), Orexigen (consultant), Organon (advisory board), Pfizer (consultant), Sanofi/Aventis (advisory board, consultant), Shering-Plough (consultant), Wyeth (consultant), and Xenoport (consultant). In addition, Dr. Keefe receives royalties from the Brief Assessment of Cognition in Schizophrenia (BACS) testing battery and the MATRICS Battery (BACS Symbol Coding).

## Authors' contributions

IAL contributed to the design of the study, and performed the statistical analysis. WD conceived the study, and contributed to its design and coordination. SKW contributed to the study design, wrote the initial draft of the manuscript, and coordinated the development of the final draft. All authors including IAL, WD, SKW, JGC, BS and RSEK participated in the analysis and interpretation of the data, and in drafting and/or revising the manuscript critically for important intellectual content. In addition, all authors read and approved the final version of the manuscript.

## Pre-publication history

The pre-publication history for this paper can be accessed here:


